# Using a Mindfulness-Based Intervention to Promote Subjective Well-Being, Trait Emotional Intelligence, Mental Health, and Resilience in Women With Fibromyalgia

**DOI:** 10.3389/fpsyg.2019.02541

**Published:** 2019-11-14

**Authors:** Javier Cejudo, Francisco-Javier García-Castillo, Pablo Luna, Débora Rodrigo-Ruiz, Roberto Feltrero, Alfonso Moreno-Gómez

**Affiliations:** ^1^Department of Psychology, Faculty of Education, University of Castilla–La Mancha, Ciudad Real, Spain; ^2^Faculty of Education, International University of La Rioja, Logroño, Spain; ^3^Salomé Ureña Higher Institute of Teacher Education, Pedagogical University, Santo Domingo, Dominican Republic

**Keywords:** fibromyalgia, mindfulness, subjective well-being, chronic pain, random assignment

## Abstract

The objective of the present study was to experimentally assess the effects of a mindfulness-based intervention (MBI) for the improvement of subjective well-being, trait emotional intelligence (TEI), mental health, and resilience in a sample of women with fibromyalgia (FM). The sample consisted of 104 women, between 29 and 77 years old (*M* = 47.59; *SD* = 5.93). The measures used were as follows: Satisfaction with Life Scale (SWLS), Positive and Negative Affection Scale (PANAS), Trait Emotional Intelligence Questionnaire Short Form (TEIQue-SF), Mental Health Questionnaire (MH-5), and Resilience Scale (ER-14). A quasi-experimental design of repeated measures with a control group (CG) was used: before and after the application of the treatment and a follow-up assessment 6 months after the completion of the intervention. In order to assess the effect of the program, the participants were randomly assigned to the experimental and control conditions. In the pretest evaluation, a multivariate analysis of variance (MANOVA) and analysis of variance (ANOVA) were carried out. In the post-test evaluation, a multivariate analysis of covariance (MANCOVA) of the study variables as a whole was performed. Then, descriptive analyses and analysis of covariance (ANCOVA) of the post-test scores (covariate pretest score) were performed. In the follow-up evaluation, a MANCOVA of the study variables as a whole was performed. Then, descriptive and ANCOVA analyses of the follow-up scores (covariate pretest score) were performed. In addition, the effect size was calculated using partial eta-squared (μ^2^). The post-test results confirmed statistically significant differences in satisfaction with life (SWL), positive affect (PA), mental health, and resilience. The follow-up results showed statistically significant differences in SWL, PA, TEI, mental health, and resilience. The study provides an effective intervention tool that has been validated experimentally. The general results allow the emphasis of the importance of the implementation of MBIs framed in non-pharmacological treatments in FM.

## Introduction

According to Häuser and Fitzcharles (2018), fibromyalgia (FM) is characterized by generalized chronic pain, unrefreshing sleep, physical exhaustion, and cognitive alterations. A more comprehensive definition refers to FM as a multifaceted disease closely related to psychological discomfort and physical pain, associated with disorders such as anxiety and depression, pathologies that, when associated with this disease, cause a worsening and irremediable chronification (Revuelta-Evrard et al., 2010). Currently, there is no consensus regarding its definition. In this way, FM has been defined as a disease characterized by chronic primary pain that is associated with emotional distress or significant functional disability (interference with activities of daily living and participation in social roles) and that cannot be explained better with another chronic pain condition (International Association for the Study of Pain [IASP], 2016). On the other hand, it was included in the *Diagnostic and Statistical Manual of Mental Disorders* (*DSM-5*) by the American Psychiatric Association [APA] (2013) as a somatic symptom disorder due to its relationship with physiopathological aspects of central sensitization, functional brain connectivity, and other changes of the central nervous system. Some authors define FM as a masked depression (Alciati et al., 2012); others define it as a persistent somatoform pain disorder (Häuser and Henningsen, 2014).

The FM diagnostic criteria were established in 1990 by the American College of Rheumatology (ACR) (Wolfe et al., 1990) and reviewed by Wolfe et al. (2010). FM is described as the existence of generalized pain of more than 3 months duration, absence of another causal pathology, and comorbidity with other syndromes and symptoms, such as chronic fatigue, unrefreshing sleep, cognitive deficit, and numerous somatic and emotional symptoms, such as anxiety and depression. Later, Wolfe et al. (2011) include a scale of FM symptoms. This scale adds the generalized pain index (WPI) and the severity of symptoms scale (SSS) to facilitate the diagnosis. Wolfe et al. (2016) published a new version of the severity scale of FM in which the doctor’s criteria are combined with the patients’ self-reports. These new criteria refined and increased the usefulness of symptom-based FM diagnosis by excluding patients with regional pain. However, they underscore the social construction of diagnosis based on symptoms and the inherent limitations in reliability and validity associated with FM criteria.

Regarding treatment, the review developed by Thieme et al. (2017), after examining the recommendations for the FM approach of some of the most influential organisms (Association of the Scientific Medical Societies in Germany, 2012; European League Against Rheumatism [EULAR], 2016), concludes that aerobic exercise, cognitive behavioral therapy, pharmacological treatment with amitriptyline, and multicomponent treatment are the most effective. In the review of recommendations for the management of FM carried out by EULAR (Macfarlane et al., 2017) through meta-analysis, relatively modest effect sizes are confirmed for most treatments. Furthermore, they add that the initial treatment must include patient education and non-pharmacological treatment, and in the case of non-effectiveness, the rest of the additional therapies should be included. A recent review of EULAR about the guidelines for the management of FM (Arumugam and MacDermid, 2019) highlights clear support for pharmacological and non-pharmacological treatment approaches in FM, considering non-pharmacological therapy as first-line treatment. Finally, EULAR (Macfarlane et al., 2017) proposes research that clarifies the individual characteristics for the administration of certain interventions, their effect in combination, the adaptation of patients to therapies, and the organization of health systems to optimize their results.

According to the therapeutic guidelines marked by the aforementioned organisms (e.g., European League Against Rheumatism [EULAR], 2016) and to the work of different authors (Glombiewski et al., 2010; Parra-Delgado et al., 2012; Van Gordon et al., 2016), the most effective treatment strategies for FM should add and integrate non-pharmacological approaches such as mindfulness, relaxation therapy, and psycho-education. Other authors highlight the importance of investigating the effects of the therapeutic application of mindfulness for FM (Aman et al., 2018; Amutio et al., 2018; Prabhakar et al., 2019).

Considering its conceptualization, the word mindfulness is the English translation of the Pali term “sati” which implies “consciousness, attention, and remembrance” (Siegel et al., 2009). Likewise, Kabat-Zinn (1990) defines it as the ability to pay attention to the experience of the present moment with a mental attitude of receptivity and acceptance.

Despite its millennial origin, it was Kabat-Zinn (1990) who introduced it to the western world, for the treatment of psychosomatic disorders, stress, and chronic pain, being aware of the great benefits that its application could bring. From these postulates, mindfulness-based interventions (MBI) emerged, specifically the Mindfulness-Based Stress Reduction (MBSR) program designed by Kabat-Zinn (1990). In addition to MBSR, others have been added, such as the Mindfulness-Based Cognitive Therapy (MBCT) program (Segal et al., 2002), which arises from the integration of mindfulness and cognitive-behavioral therapy for depression, and others like MBEating (MBE) (Kristeller and Wolever, 2011). Many studies support the integration of mindfulness into health care as part of self-care and the management of different diseases (Greeson and Chin, 2019).

On the other hand, it has been observed that MBIs are effective in primary care, especially for patients with symptoms of stress, anxiety, or depression (Hervás et al., 2016). Authors such as Vásquez-Dextre (2016) expose clinical reasons for applying mindfulness in mental and physical health problems. Besides, the growing interest in MBI has been caused by its effectiveness in increasing the well-being of individuals by improving some physical and psychological aspects (Hervás et al., 2016). MBIs seem to enhance the use of positive reevaluation (e.g., Carmody and Baer, 2008). Also, some studies have shown that with MBI, in a population with depressive symptoms, there is a positive interrelation between positive affect (PA) [an affective component of subjective well-being (AWB)] and positive cognitions [a cognitive component of subjective well-being (CWB)] (Garland et al., 2015).

Various meta-analyses (Hilton et al., 2017; Zou et al., 2018) highlight the positive effects of mindfulness in major depression disorder, associated with significant improvements in depression, quality of life related to physical health and mental health. Also, they recognize positive effects on health in general, in comparison to control groups (CGs). There are also works that advise the use of mindfulness in anxiety and stress disorders (Gallegos et al., 2013; De Frias and Whyne, 2015) considering these interventions as a viable protective factor to reduce the dangers of stress, reducing the reactivity to the stressor. Therefore, it could serve as a protective psychological process for health (Yat Ho Li and Bressington, 2019).

Similarly, research on the impact of the practice of mindfulness in chronic pain is increasing, and there are works with positive results in coping with it (McCraken and Vowles, 2014). Other works conclude that the practice of mindfulness improves postural awareness, causing a decrease in pain in patients with spinal pain and shoulder pain (Cramer et al., 2018) and that training in mindfulness helps relieve the suffering associated with chronic pain, improving its management and the neurobiological mechanisms involved (Brown et al., 2015).

Specifically in the field of study on FM, there is evidence that MBI is beneficial for treating certain symptoms, such as depression, anxiety, anger, and poor quality of life (Grossman et al., 2007; Sephton et al., 2007; Schmidt et al., 2011; Davis and Zautra, 2013; Amutio et al., 2015). Other authors such as Van Gordon et al. (2017) claim that in patients with FM through an 8-week MBI, they found benefits in pain perception, sleep quality, and psychological distress. Furthermore, these effects were maintained 6 months after the conclusion of the treatment. After applying an 8-week MBI in women with FM, Cash et al. (2015) conclude significant improvements in some of their associated symptoms, such as fatigue, stress, sleep, pain, salivary cortisol, and overall well-being. Also, these benefits were maintained in the follow-up evaluation.

In the same direction, Davis et al. (2015) after developing an MBI in patients with FM, with randomized study, revealed improvements in social functioning, PA, and effectiveness in coping with pain and stress. Besides, patients with depressive symptoms reported improvements in loneliness, family stress, and PA. Likewise, Schmidt et al. (2011), through a randomized clinical study in patients with FM, confirm that participants assigned to the MBSR did not report significant reductions in pain, but improvements in quality of life compared to the CG. However, these benefits were not maintained at follow-up (2 months), which suggests that MBSR does not produce stabilized improvements in the quality of life of patients with FM. Grossman et al. (2007), through a randomized clinical study in women with FM, obtained improvements in pain perception, quality of life, pain management, anxiety, depression, and somatic complaints. These results were maintained in the follow-up evaluation at 3 years. Quintana and Rincón-Fernández (2011) through an MBI with women with FM found significant improvements in quality of life, vitality, and mental health. Likewise, the participants reported improvements in the presence and intensity of pain, in coping strategies and perceptions in general health. Lakhan and Schofield (2013) conducted a systematic review of MBI in the treatment of somatic disorders (including FM) and conclude that these interventions are effective in reducing pain, depression symptoms, and anxiety symptoms – improving the quality of life in these patients.

From other approaches, Amutio et al. (2015), through an MBI with a duration of 7 weeks in patients with FM, corroborate reductions in symptoms of anxiety, depression, and improvements in the ability of participants to regulate their anger. These results were maintained in a follow-up at 3 months. Amutio et al. (2018) showed that, compared to the control condition, MBI was effective in reducing insomnia and improving sleep quality in line with the studies of Kanen et al. (2015). It is important to note that several authors state that MBI produces more stabilized improvements in measures of pain and quality of life as a function of frequency, continuity of practice, and experience of meditation training (Quintana and Rincón-Fernández, 2011; Adler-Neal and Zeidan, 2017). They also point out that the daily average of mindfulness practice is a significant predictor of changes in all outcome variables (Van Gordon et al., 2017).

This work is projected with the idea of providing greater consistency to the application of non-pharmacological therapies in the treatment of FM, specifically the use of MBI. Thus, the purpose of the present study was to evaluate the effects of an MBI on subjective well-being, trait emotional intelligence (TEI), mental health, and resilience in women with FM. It was hypothesized that compared to the waitlist CG, women with FM that completed the mindfulness intervention would demonstrate significant improvements in the variables mentioned above, both in the post-test phase and in the follow-up phase (6 months).

## Materials and Methods

### Design

A randomized experimental design was conducted with three repeated measures (pretest, post-test, and a 6-month follow-up). The participants were randomly assigned either to the experimental group (EG) or to the usual treatment of the CG. The usual treatment of the CG was focused on psychoeducation and included information on common symptoms in FM and advice on self-care. This treatment was performed by the psychologist of the FM association. All this was done in addition to the pharmacological treatment for pain indicated by specialists.

### Participants

A total of 132 women from the Association of Relatives and Affected by Fibromyalgia of the province of Ciudad Real (Spain) voluntarily participated in the investigation. To participate in the study, participants had to meet three *inclusion criteria*: (a) be diagnosed with FM syndrome (e.g., via a letter from a doctor of pain consultant), (b) commit to the daily practice of mindfulness, and (c) not be currently receiving mindfulness training. Two *exclusion criteria* were also established: (a) be diagnosed with a mental disorder and (b) receive individual psychological therapy. An adjusted sample size of 102 participants was estimated using GPower 3.1.9.2 software (Faul et al., 2007) for the hypothesis contrast for independent samples (*p* < 0.05).

**FIGURE 1:**
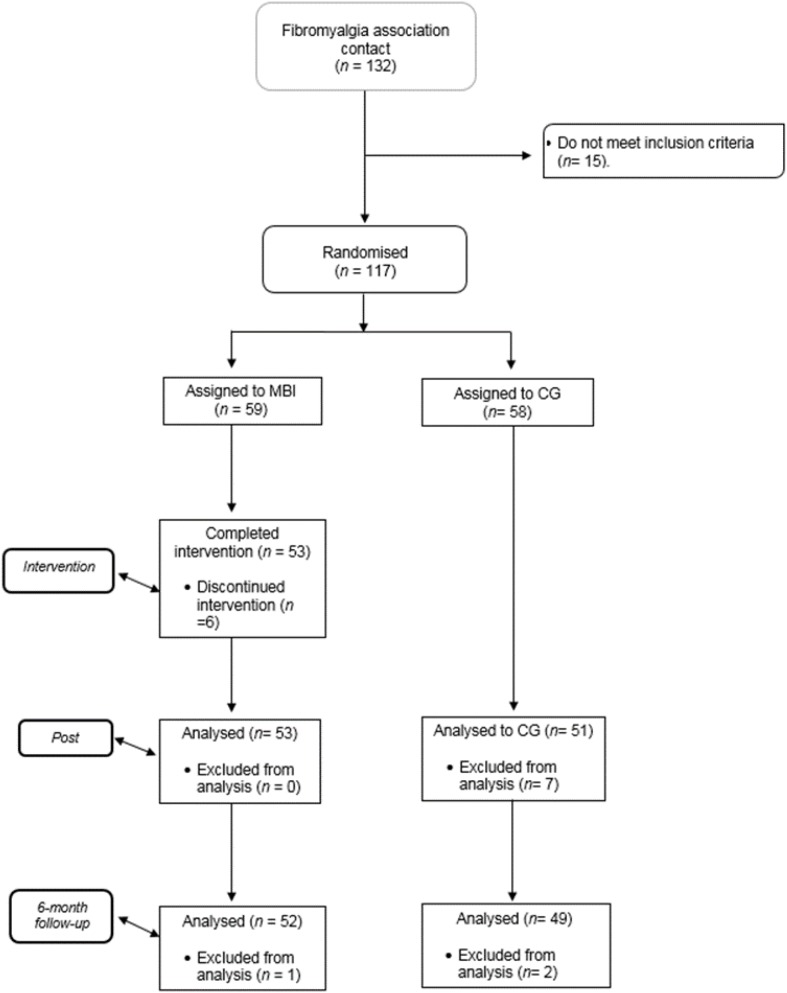
Participant flow diagram. MBI mindfulness-based cognitive therapy (EG), treatment as usual control group condition (CG).

The 117 participants who met the proposed criteria were randomly assigned to the EG of MBI (*n* = 59) or the usual treatment of the CG (*n* = 58) using the Random Number Generator program (Devilly, 2004). The therapy was completed by 104 of the 117 patients. The dropout rate in the EG was 16.95% if it is considered that the experimental mortality is the lack of attendance of at least 50% of the sessions of the program. Participant flow is displayed below (refer to Figure 1).

The sample consists of 104 women, aged 29–57 (*M* = 47.59; *SD* = 5.93). A non-probabilistic sampling was used, but subjects were assigned at random to the experimental (*n* = 53) and control (*n* = 51) condition. The age differences in the two conditions were not significant, χ^2^ = 1.79, *p* > 0.05.

Regarding the demographic characteristics; 87.5% were married, 10.5% divorced, and 2% were widows. Concerning the level of studies of the participants; 63.1% had completed primary education, 23.2% secondary education, and 13.7% university studies; 15.2% of patients took antidepressants, 25.4% took anxiolytics, and 11.9% took both types of medication.

### Measures

In this study, we used well-established measures with appropriate psychometric properties (refer to Table 1).

**TABLE 1:** Reliability evidence.

	**α**	**CR**	**AVE**	**Ω**
SWLS	0.90	0.88	0.659	0.92
Positive affect	0.89	0.84	0.541	0.84
Negative affect	0.81	0.77	0.533	0.81
TEIQue-SF	0.88	0.92	0.617	0.91
MH-5	0.78	0.83	0.597	0.80
ER-14	0.77	0.79	0.502	0.78

*α, Cronbach’s alpha; CR, composite reliability; AVE, average variance extracted; Ω, McDonald’s omega index.*

#### Satisfaction With Life Scale (SWLS) (Diener, 1984)

In this study, the version adapted to Spanish has been used (Vázquez et al., 2013). This scale is composed of five items, in which participants are expected to indicate the degree of agreement with each statement using a seven-point Likert scale (from 1 = *strongly disagree* to 7 = *strongly agree*). Diener (1984) states that this scale evaluates the cognitive CWB referring to the result of the evaluation of the processing of the information that people make of their lives.

#### Positive and Negative Affection Scale (PANAS) (Watson et al., 1988)

A Spanish version (Sandín, 2003) was used. PANAS is a self-reported adjective checklist designed for the assessment of 20 different feelings and emotions. It contains two subscales each with 10 items, representing two constructs: PA and negative affect (NA). Participants used a five-point scale (from 1 = *very slightly or not at all* to 5 = *extremely*). This scale evaluates the AWB. According to Diener (1984), the AWB implies an individual hedonistic balance, that is, the frequency with which people experience emotions of a positive and negative nature.

#### Trait Emotional Intelligence Questionnaire Short Form (TEIQue-SF) (Petrides, 2009)

A Spanish version (Pérez, 2003) was used. The TEIQue-SF is a self-report inventory designed to measure global TEI with 30 items using seven-point Likert scale response options (from 1 = *completely disagree* to 7 = *completely agree*). This measure provides a total score that is obtained by adding the scores from the 30 items.

#### Mental Health Scale (MH5) (Ware and Sherbourne, 1992; Adapted to Spanish by Alonso et al., 1995)

The Mental Health-5 (MH-5) is one of the subscales of the SF-36 health questionnaire by Ware and Sherbourne (1992). The MH-5 is composed of five items on emotional well-being. A high score on this scale is associated with better mental health. The questions included are similar to: “*During the past four weeks, how long were you very nervous?*” The answers are encoded using a six-point Likert scale (from 1 = *always* to 6 = *never*).

#### Resilience Scale (ER-14) (Wagnild, 2009; Adapted to Spanish by Sánchez-Teruel and Robles-Bello, 2015)

It consists of 14 items. The answers are coded through a seven-point Likert scale (from 1 = *strongly disagree* to 7 = *strongly agree)*. It measures the degree of individual resilience, considered a positive psychological characteristic that allows the individual to adapt to adverse situations. Also, this scale presents negative and significant correlations with depression and anxiety (e.g., Nishi et al., 2010).

### Procedure

The study followed a quasi-experimental design of repeated measures (pretest and post-test) including a CG, where the following variables were assessed: satisfaction with life (SWL), PA, and NA, mental health (MH), TEI, and resilience (RS).

### Ethical Considerations

All participants gave their informed consent, and the study was approved by the board of the association involved. Confidentiality and anonymity were guaranteed in order to comply with the Law on Protection of Personal Data of the Ethics Committee for Research on Human Beings (CEISH). The international guidelines for studies with human subjects described in the Nuremberg Code and the Declaration of Helsinki were applied. After completing all phases of the evaluation, the MBI was carried out with the CG participants.

### Training Program Description

An MBI was carried out based on some previous works (e.g., Parra-Delgado et al., 2012; Amutio et al., 2015). This MBI is structured in two parts, in line with some of the principles of MBSR (Kabat-Zinn, 1990; Schmidt et al., 2011): (a) formal practice carried out in groups during face-to-face sessions and (b) the informal practice carried out individually at home through audio-guide. Therefore, we can say that the present intervention is MBSR-adapted.

The formal practice consisted of 20 group sessions. These sessions were held in 20 weeks (that is, one per week), with a duration of 1 h. The training program included three content blocks – (1) mindfulness meditation techniques according to some previous works (Kabat-Zinn, 1990; Schmidt et al., 2011), (2) exhibition and debate on the exercises used in the formal practical session, and (3) Vipassana meditation (Hart, 1994) – that promote values such as impermanence, compassion, acceptance, forgiveness, and detachment with reference to the work done by Amutio et al. (2015).

Similarly, the present MBI attempts to conform to the criteria of Van Gordon et al. (2017) on Interventions Based on Second Generation Comprehensive Care (SG-MBIs). These interventions emphasize and recognize the spiritual aspect of mindfulness. In addition, Van Gordon et al. (2015) highlight the possible positive effects of SG-MBIs in the treatment of FM.

The main objective of this MBI is to improve the relationship of participants with pain by taking perspective on the intrusive, ruminant, persistent, and egodystonic thoughts that usually accompany pain, focusing attention toward more adaptive emotional states in order to accept the experience of pain.

Each weekly session presents the following structure: (1) reflection on the practical exercises carried out during the previous week for 10 min, (2) body scan (10 min), (3) presentation of the exercises and explanation of their meaning for each session (20 min), and (4) meditative practice for 20 min.

The informal practice was carried out at home with the help of an audio-guide. Also, the participants were encouraged to practice body scan for 5 min and attention focused on breathing (15 min). The informal practice was carried out during active treatment, as well as during the follow-up phase.

The program was designed and developed by an instructor with extensive experience in the practice and teaching of mindfulness techniques in the field of health. Program sessions are detailed in Table 2.

**TABLE 2:** Intervention program sessions.

**Session**	**Objectives**	**Contents**
1–3	Know mindfulness techniques	– Definition and analysis of the term by different authors – Mindfulness technique, status, and via
4–9	Know/develop the types of practice; formal and informal	– Explanation of the types of formal and informal practice with attitude (curiosity, openness, and no judgment) – Commitment to practice – Postural adjustments: lying, sitting, standing, and walking
10–14	Practice calm/relaxation	– Awareness about the body (weight/calmness)
15–18	Identify and observe breathing	– Start, breathing characteristics, frequency, changing character, and parts (inspiration, expiration, and apneas) – Awareness of weight, associated with calm and breathing
19–20	Cultivate full attention	– Attention on breathing – Focused attention – Open monitoring – Vipassana meditation (compassion, acceptance, forgiveness, and detachment)

### Statistical Analysis

Initially, the normality of the study variables was tested with a Kolmogorov–Smirnov test. All the variables were adjusted to the assumption of normality. The analyses were conducted with a confidence interval of 95%. After, reliability coefficient Cronbach’s alpha (α), composite reliability (CR), average variance extracted (AVE), and McDonald’s omega coefficient (Ω) were calculated to obtain reliability evidence. First, a multivariate analysis of variance (MANOVA) was performed with total pretest scores from the variables included in the study in order to confirm the possible pretest difference in the variables, as a whole, between EG participants and CG participants. Second, in order to determine the program’s effect, descriptive analysis (mean and standard deviations) and repeated-measures analysis of variance (ANOVA) were carried out with each one of the scores obtained for the instruments used during the pretest phase. Third, having confirmed the homogeneity of the two groups *a priori*, and in order to determine whether the change was significantly different in the EG versus CG participants, a multivariate analysis of covariance (MANCOVA) was performed on the study’s variables as a whole.

Besides that, descriptive analyses and analyses of covariance were performed on post-test scores [post-test analysis of covariances (ANCOVAs) covarying for pretest scores]. In the follow-up evaluation, a MANCOVA of the study variables as a whole was performed. Then, descriptive and covariance analyses of the follow-up scores were conducted (follow-up ANCOVAs, covarying for pretest scores). The effect size (μ^2^) of the differences was calculated using partial eta-squared (Tabachnick and Fidell, 2007). The effect size was analyzed based on four ranges: 0–0.009, *negligible*; 0.010–0.089, *low-effect size*; 0.090–0.249, *medium-effect size*; and >0.250, *big-effect size*.

## Results

Results obtained in the pretest or basal evaluations are first presented, followed by the results for evaluating the impact of the MBI in the variables studied (post-test). Finally, the results obtained in the follow-up are collected.

**FIGURE 2:**
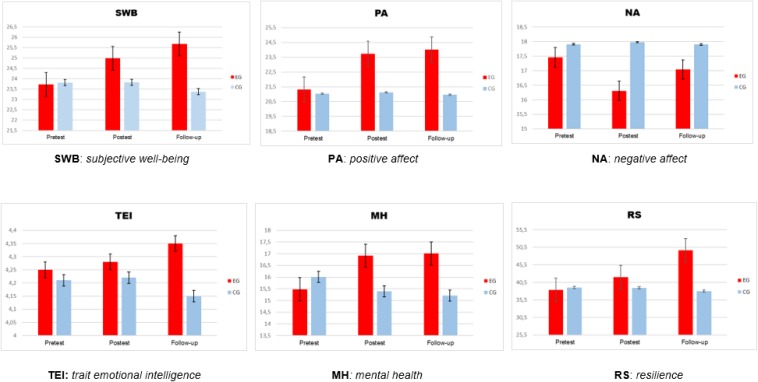
Effects of MBI on the variables of the study across three times (pretest, before training; post-test, after training; follow-up, 6 months after training).

The pretest MANOVA results did not reveal statistically significant differences between the groups prior to the intervention, Wilks’ Lambda, Λ = 0.739; *F*(5,99) = 0.628; *p* = 0.249, with a small effect size (μ^2^ = 0.030, *r* = 0.11). The results of the ANOVA in the pretest phase (refer to Table 3) showed that before starting the program, there were no statistically significant differences in any of the variables dependent on the study (refer to Figure 2).

**TABLE 3:** Averages and standard deviations in the variables under study (subjective well-being, trait emotional intelligence, mental health, and resilience) in the experimental and control groups.

	**Pretest**					**Post-test**					**Follow-up**				
			
	**EG**	**CG**				**EG**	**CG**				**EG**	**CG**			
	***M* (*SD*)**	***M* (*SD*)**	***F***	***p***	**μ^2^**	***M* (*SD*)**	***M* (*SD*)**	***F***	***p***	**μ^2^**	***M* (*SD*)**	***M* (*SD*)**	***F***	***p***	**μ^2^**
SWL	23.72 (5.91)	23.81 (5.85)	0.265	0.469	0.003	24.98 (5.62)	23.82 (5.96)	1.384	0.041	0.015	25.67 (5.02)	23.37 (6.03)	2.984	0.009	0.143
PA	21.32 (5.04)	21.03 (5.24)	0.071	0.981	0.001	23.73 (4.92)	21.12 (5.61)	2.127	0.026	0.032	24.01 (4.63)	20.96 (6.12)	2.872	0.041	0.028
NA	17.46 (4.56)	17.91 (4.76)	0.184	0.719	0.001	16.31 (4.32)	17.98 (4.81)	0.631	0.139	0.014	17.04 (5.21)	17.90 (5.11)	0.995	0.532	0.009
TEI	4.25 (0.85)	4.21 (0.89)	0.228	0.641	0.000	4.28 (0.74)	4.22 (0.87)	1.831	0.241	0.003	4.35 (0.62)	4.15 (0.81)	4.126	0.037	0.083
MH	15.48 (5.29)	16.01 (4.99)	2.372	0.077	0.002	16.92 (3.32)	15.39 (4.95)	2.516	0.028	0.021	17.01 (3.42)	15.21 (4.99)	2.351	0.037	0.022
RS	38.53 (7.12)	39.04 (7.01)	0.993	0.164	0.005	41.98 (6.96)	38.92 (6.84)	1.749	0.012	0.043	49.65 (8.01)	37.99 (7.03)	3.768	0.003	0.128

*SWL, satisfaction with life; PA, positive affect; NA, negative affect; TEI, trait emotional intelligence; MH, mental health; RS, resilience; μ^2^, eta squared effect size; EG, experimental group (*n* = 59); CG, control group (*n* = 58); *M*, means; *SD*, standard deviation.*

### Post-test Evaluation

Results from the pretest–post-test MANCOVA revealed significant differences between the two conditions, Wilks’ lambda, Λ = 1.526; *F*(5,99) = 3.214; *p* = 0.007, with an average effect size (μ^2^ = 0.199, *r* = 0.33).

#### Effects on Subjective Well-Being

The results showed significant improvements in life satisfaction in favor of EG (refer to Table 3). The size of the effect (eta-squared) reported low-effect size (μ^2^ = 0.015).

The results of the analyses showed a significant increase in PA scores in favor of the EG. The size of the effect was a low-effect size for PA (μ^2^ = 0.032). However, a significant decrease in NA is not confirmed. It is necessary to indicate that there is no significant relationship between PA and NA (*r* = -0.125, *p* = 0.144).

#### Effects on Trait Emotional Intelligence

Regarding the variable TEI, the results did not confirm significant improvements in favor of the EG.

#### Effects on Mental Health

Regarding the variable mental health, the results analyzed showed significant improvements in favor of the EG. The size of the effect was low-effect size (μ^2^ = 0.021).

#### Effects on Resilience

Finally, regarding the resilience variable, the results analyzed showed significant improvements in favor of the EG. The size of the effect was low-effect size (μ^2^ = 0.043).

### Follow-Up (6 Months)

Results from the pretest follow-up MANCOVA revealed significant differences between the two conditions, Wilks’ lambda, Λ = 1.168; *F*(5,96) = 3.581; *p* = 0.009, with an average effect size (μ^2^ = 0.298, *r* = 0.27).

#### Effects on Subjective Well-Being

The results showed significant improvements in SWL in favor of EG (refer to Table 3). The size of the effect was medium-effect size (μ^2^ = 0.143).

Results showed a significant increase in PA scores in favor of EG, without showing a decrease in NA. The size of the effect in PA was low-effect size (μ^2^ = 0.028).

#### Effects on Trait Emotional Intelligence

Concerning the variable TEI, the results confirmed significant improvements in favor of the EG. The size of the effect was low-effect size (μ^2^ = 0.083).

#### Effects on Mental Health

Regarding the variable mental health, the results analyzed showed significant improvements in favor of the EG. The size of the effect was low-effect size (μ^2^ = 0.022).

#### Effects on Resilience

Finally, regarding the resilience variable, the results analyzed showed significant improvements in favor of the EG. The size of the effect was medium-effect size (μ^2^ = 0.128).

## Discussion

The present study analyzes the effects of an MBI on subjective well-being, TEI, mental health, and resilience in a sample of women with FM. In this sense, Van Gordon et al. (2015) emphasize that, despite the increasing amount of literature on the effects of MBI, it is necessary to continue investigating the effectiveness of these interventions in FM.

The results showed statistically significant improvements among the women who received the intervention compared to those who did not participate. The effects of the MBI in the post-test, on the EG in comparison with CG show the following results: (1) significant increase in SWL (CWB), (2) significant improvement of PA but without a decrease in NA (AWB), (3) no improvement in TEI score, (4) significant increase in mental health, and (5) significant increase in resilience. On the other hand, the effects of the MBI in the follow-up show the following results: (1) significant increase in SWL (CWB), (2) significant improvement of PA but without a decrease in NA (AWB), (3) improvement in the score TEI, (4) significant increase in mental health, and (5) significant increase in resilience.

First, the results show partial improvements in some of the components of subjective well-being. In this sense, the results in the post-test and in the follow-up show an improvement in the SWL, that is to say, according to Diener (1984), improvement of the evaluation of the processing of information that people make of their lives (CWB). The results are consistent with those found in other studies that have demonstrated the efficacy of MBI to improve subjective well-being (Grossman et al., 2007; Quintana and Rincón-Fernández, 2011; Schmidt et al., 2011; Lakhan and Schofield, 2013; Cash et al., 2015; Davis et al., 2015). These results may likely be due to MBI modifying the perception that people with FM have of the negative emotional symptoms caused by pain, facilitating greater subjective well-being (Grossman et al., 2007; Sephton et al., 2007). Concerning the AWB, the results in the post-test and in the follow-up corroborate an improvement in PA. However, a decrease in NA is not obtained in congruence with various studies (e.g., Sephton et al., 2007; Davis and Zautra, 2013; Davis et al., 2015). In this sense, these results reinforce the conclusions of Watson et al. (1988) in that both factors (PA and NA) constitute two independent dimensions of affect and, therefore, are not correlated with each other. These improvements likely derive from the strong relationship between PA and quality of life related to health (e.g., Ong, 2010). A possible explanation for these results could be that PA presents more strong relationships than NA and, therefore, assumes a more relevant position in physical and psychological health (e.g., Fredrickson, 2001; Cohen et al., 2006). Likewise, MBI can be effective in disconnecting the NA response that accompanies pain, promoting subjective well-being (Sephton et al., 2007). The improvement of PA after an MBI is consistent with the results found by other larger studies with a non-clinical population (e.g., Orzech et al., 2009) as well as in the clinical population (e.g., Geschwind et al., 2011). On the other hand, one of the contents of this MBI is compassion. We consider that it can be an important factor to explain the reduction of certain negative symptomatology as well as the improvement of subjective well-being (Hervás et al., 2016).

Second, the results confirm that there are no significant improvements in the post-test concerning TEI. On the contrary, the results confirm an improvement of TEI in the follow-up. From our point of view, it is likely that the direct relationship between the practice of mindfulness and some of the dimensions of emotional intelligence, such as clarity and emotional repair, may have some influence (De la Fuente et al., 2010), as well as emotional regulation (Huang et al., 2019). On the other hand, some authors (Rodríguez-Ledo et al., 2018) have found positive relationships between the capacities of mindfulness and emotional intelligence. Rodríguez-Ledo et al. (2018) point out that individuals who practice mindfulness present higher scores in intrapersonal competences (e.g., emotional self-perception or emotional self-regulation) and interpersonal competences (e.g., empathy, emotional regulation of others or coping with life’s adverse situations) that compound the emotional intelligence construct. In our opinion, an interesting aspect of the results obtained is the appearance of an improvement in the TEI in the follow-up. Thus, if TEI is defined as a constellation of traits related to the typical way an individual processes information of an emotional nature and react to emotional situations, it is likely that individuals need some time to recognize these typical patterns in their habitual behavior.

Third, the results show improvements in mental health in the post-test and in the follow-up. Our results are in line with other studies that have studied the effects of MBI, mainly in depression and anxiety, negatively related to mental health (Sephton et al., 2007; Quintana and Rincón-Fernández, 2011; Parra-Delgado et al., 2012; Lakhan and Schofield, 2013; Amutio et al., 2015; Van Gordon et al., 2017). The positive effects of MBI on mental health can be explained by the influence of these interventions on the improvement that takes place in the automatic processing of emotion, that is, a lower emotional reactivity to negative emotions (Hervás et al., 2016). In this sense, it is likely that the MBI seems to promote the use of more adaptive emotional regulation strategies, such as positive reevaluation, thus improving the mental health of individuals (e.g., Carmody and Baer, 2008). In addition, MBIs seem to reduce the use of maladaptive emotional regulation strategies, such as rumination and catastrophism, that negatively impact the mental health of individuals (Ortner et al., 2007). An unexpected result was that the present MBI did not result in a decrease in NA while it did improve the mental health of the participants. In this regard, it is pertinent to indicate that the results of some studies show stronger relationships between PA and mental health than the relationships between NA and mental health (Vera-Villarroel and Celis-Atenas, 2014). Furthermore, the evidence suggests that PA influences pain and adaptive coping strategies, over and above the influence of NA (Finan and Garland, 2015).

Finally, as regards the resilience variable, the results show an improvement in resilience in the post-test and in the follow-up. As we have previously stated, resilience is defined as a positive personality characteristic that allows the individual to adapt to adverse situations (Wagnild, 2009). In this sense, we share the idea that among the processes that explain these positive changes in resilience is the influence on acceptance training (included in the present MBI), since it cushions the individual against the impact of life’s difficult situations (e.g., Hervás, 2011). We agree with some authors (Hervás et al., 2016) that MBI offers participants a broader repertoire of cognitive and behavioral resources to be effective in situations of a stressful nature.

### Limitations and Future Directions

It is important to note some limitations of the present investigation; first, regarding the generalization of the results, since the sample is composed only of women affected by FM, it would be necessary to investigate the effects of MBI on men who are diagnosed with FM due to the unique characteristics they have in these interventions (Kanen et al., 2015). On the other hand, it would be necessary to replicate the experience with more heterogeneous socially and culturally samples. In summary, with these promising results, additional research with MBI is required to extend these findings and test their application. Second, it is necessary to point out that only self-report measures have been used; it would be necessary to support these findings with other measures. However, the data collected with self-reports are related to neuroimaging measures, of a more objective nature (Brewer et al., 2011).

Third, another limitation of the present study, and in general of the MBI, is the difficulty to make comparisons between the results of the different studies due to the heterogeneity of the different MBI (Kanen et al., 2015). This implies that research to promote the effectiveness of MBI should be interpreted with caution given the existing differences in its design, development, and evaluation.

Regarding the future lines of research, the evaluation of the effect of MBI on other variables can be suggested, for example, perception of pain. In this sense, some studies have shown improvements in the perception of pain (e.g., Grossman et al., 2007; Davis et al., 2015); however, other studies do not support these findings (e.g., Schmidt et al., 2011). On the other hand, research should continue on the optimal level of the duration of the MBI, the contents that include the different MBI (Kanen et al., 2015) or the training of MBI instructors (Hervás et al., 2016). Nevertheless, it is necessary to investigate the possible adverse effects of MBI, as some authors warn (e.g., Shapiro, 1992; Didonna and Gonzalez, 2009). Furthermore, we believe that future research should include the analysis of the effects of MBI on the impact of FM on functional capacity and quality of life, through specific instruments such as the Fibromyalgia Impact Questionnaire (FIQ) (Martín et al., 2014).

## Conclusion

Given these results, we wish to emphasize that the results obtained in the present investigation suppose empirical support to the use of MBI in women with FM. Also, it is necessary to highlight the importance of implementing MBI in patients with FM to promote subjective well-being, TEI, mental health, and resilience that can act as protective psychological resources that help people cope effective daily demands. We share with some authors that these interventions present a relatively low risk, and therefore, the FM treatment protocols should include this line of treatment (Cash et al., 2015).

Also, the present study can enrich the research on the effects of MBI in people with FM, since maintaining the structure of the MBSR (Kabat-Zinn, 1990; Schmidt et al., 2011) intends to make an adaptation to the characteristics of this collective.

## Data Availability Statement

All datasets generated for this study are included in the article/supplementary material.

## Ethics Statement

The studies involving human participants were reviewed and approved by the Ethics Committee for Research on Human Beings, University of Castilla–La Mancha. Patients/participants gave written informed consent to participate in this study. The animal study was reviewed and approved by the National Institutes of Health Animal Care and Use Guidelines.

## Author Contributions

Each author has made substantial contributions to the work. JC, F-JG-C, and AM-G conceived or designed the work. RF was responsible for audio-guide design. JC and F-JG-C collected data and drafted the manuscript. JC and PL were responsible for data analysis and interpretation. PL, DR-R, and RF were responsible for critical revision of the manuscript. JC, F-JG-C, PL, DR-R, RF, and AM-G approved the final version of the manuscript to be published.

## Conflict of Interest

The authors declare that the research was conducted in the absence of any commercial or financial relationships that could be construed as a potential conflict of interest.
